# miR-206 regulates cisplatin resistance and EMT in human lung adenocarcinoma cells partly by targeting MET

**DOI:** 10.18632/oncotarget.8229

**Published:** 2016-03-21

**Authors:** Qing-yong Chen, De-min Jiao, Jian Wang, Huizhen Hu, Xiali Tang, Jun Chen, Hao Mou, Wei Lu

**Affiliations:** ^1^ Department of Respiratory Disease, The 117th Hospital of PLA, Hangzhou, Zhejiang, 310013, P.R. China; ^2^ Department of Oncology, The 117th Hospital of PLA, Hangzhou, Zhejiang, 310013, P.R. China

**Keywords:** miR-206, cisplatin resistance, epithelial-mesenchymal transition (EMT), MET, lung adenocarcinoma

## Abstract

MicroRNAs (miRNAs) play a critical role in drug resistance and epithelial-mesenchymal transition (EMT). The aims of this study were to explore the potential role of miR-206 in governing cisplatin resistance and EMT in lung cancer cells. We found that both lung adenocarcinoma A549 cisplatin-resistant cells (A549/DDP) and H1299 cisplatin-resistant cells (H1299/DDP) acquired mesenchymal features and were along with low expression of miR-206 and high migration and invasion abilities. Ectopic expression of miR-206 mimics inhibited cisplatin resistance, reversed the EMT phenotype, decreased the migration and invasion in these DDP-resistant cells. In contrast, miR-206 inhibitors increased cisplatin resistance, EMT, cell migration and invasion in non-DDP-resistant cells. Furthermore, we found that MET is the direct target of miR-206 in lung cancer cells. Knockdown of MET exhibited an EMT and DDP resistant inhibitory effect on DDP-resistant cells. Conversely, overexpression of MET in non-DDP- resistant cells produced a promoting effect on cell EMT and DDP resistance. In lung adenocarcinoma tissues, we demonstrated that low expression of miR-206 were also correlated with increased cisplatin resistance and MET expression. In addition, we revealed that miR-206 overexpression reduced cisplatin resistance and EMT in DDP-resistant cells, partly due to inactivation of MET/PI3K/AKT/mTOR signaling pathway, and subsequent downregulation of MDR1, ZEB1 and Snail expression. Finally, we found that miR-206 could also sensitize A549/DDP cells to cisplatin in mice model. Taken together, our study implied that activation of miR-206 or inactivation of its target gene pathway could serve as a novel approach to reverse cisplatin resistance in lung adenocarcinomas cells.

## INTRODUCTION

Lung cancer, predominantly non-small cell lung cancer (NSCLC), is the leading cause of cancer-related mortality worldwide. Patients with NSCLC are mostly treated with platinum-based chemotherapy in combination with radiation therapy. However, the development of chemoresistance, either intrinsic or acquired, is a major obstacle limiting successful treatment [1]. Cisplatin (DDP) is still one of the commonly used chemotherapeutic agents against lung cancer due to its therapeutic advantages, such as high efficiency, mild side effects and easy administration. However, cisplatin resistance often occurs in clinical practice [2]. Thus, adjuvant therapy to enhance cisplatin efficiency becomes an important chemotherapeutic strategy.

Accumulating studies indicate that there are several major mechanisms of drug resistance in cancer cells, such as increased detoxification of anticancer drugs by glutathione system, defective apoptosis pathway, increased levels of DNA repair or DNA tolerance, decreased uptake of water-soluble drugs and enhanced drug efflux from cancer cells mediated by ATP-binding cassette (ABC) transporters [3–5]. Several studies showed that the drug-resistant cancer cells display features of epithelial-mesenchymal transition (EMT), which is defined by the loss of intracellular links along with the gain of migratory and invasive abilities [6, 7]. Specifically, cells with decreased expression of the epithelial marker E-cadherin and increased expression of mesenchymal molecules including Snail/Snai1, Slug/Snai2, Vimentin, zinc-finger E-box binding homeobox 1 (ZEB1), lead to enhanced motility, invasion and drug resistance [8]. In addition, PI3K/AKT/mammalian target of rapamycin (mTOR) signaling has also found to confer resistance to DDP-based treatment in many cancers [9, 10]. Furthermore, it has been reported that there exist a cross-talk between EMT programming and PI3K/AKT/mTOR pathway [11].

The microRNAs (miRNAs) are a group of small non-coding RNAs. The basic mechanism of miRNA action is that miRNA could bind to the 3'UTR of target mRNAs, resulting in translational repression or target mRNA cleavage [12]. Recent studies suggested that the acquisition of drug resistance by cancer cells might be modulated via the changes in miRNA levels [13, 14]. For instance, miR-135a/b are downregulated in cisplatin resistance (A549/DDP) cells, and the overexpression of miR-135a/b sensitizes A549/DDP cells to cisplatin by targeting MCL1 (myeloid cell leukemia 1) [15]. Up-regulation of miR-27a can suppress RKIP (Raf kinase inhibitory protein) expression and in turn contribute to chemoresistance of lung adenocarcinoma cells to cisplatin [16]. Upregulation of miR-451 expression inactivates the AKT signaling pathway and enhanced cisplatin induced apoptosis in A549 cells [17]. MiR-513a-3p can sensitize human lung adenocarcinoma cells to cisplatin by targeting GSTP1 (Glutathione S-transferase P1)[18]. MiR-92b is significantly up-regulated in lung cancer cells and knockdown of miR-92b inhibits cell growth and sensitizes the A549/DDP cells to DDP by target PTEN (phosphatase and tensin homolog) [19].

MiR-206 is one of the most studied and best characterized miRNAs to date, which specifically expressed in skeletal muscle [20]. Recently, there has been increasing interest in understanding the role of miR-206 in cancer, and down-regulation of miR-206 has been observed in different types of cancers [21–27]. Decreased expression of miR-206 in gastric cancer is associated with tumor progression and poor survival [24]. miR-206 suppresses breast cancer cell migration and invasion by targeting Cdc42 [28]. miR-206 can inhibit the expression of VEGF and regulate the apoptosis and migration of laryngeal cancer cells [29]. We and others have also reported miR-206 overexpression could inhibit invasion of lung cancers [22, 26, 30]. However, whether miR-206 is involved in regulating cisplatin resistance and EMT in human lung adenocarcinomas remains unclear.

In this study, we found miR-206 was down-regulated in both A549/DDP cells and H1299/DDP cells. Overexpression of miR-206 or knockdown of its target MET reversed the mesenchymal features and sensitized DDP-resistant cells to cisplatin. More importantly, we demonstrated that decreased miR-206 levels induced cisplatin resistance and EMT phenotype due to activation of MET/PI3K/AKT/mTOR axis, upregulation of MDR1, ZEB1 and Snail expression in DDP-resistant cells. These results provide new insights into the molecular mechanisms of cisplatin resistance induced by decreased miR-206 levels in lung adenocarcinoma cells and suggest miR-206 and its target gene pathway could be novel therapeutic targets to reverse cisplatin resistance of lung adenocarcinoma cells.

## RESULTS

### Cisplatin resistant lung adenocarcinoma cells exhibit EMT features and have enhanced MDR1 expression

**Figure 1:**
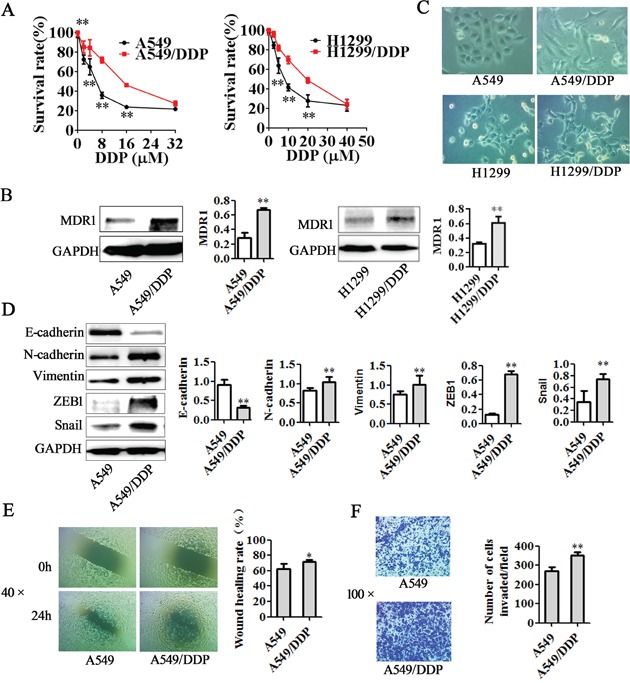
Differences between DDP-resistant cells and non-DDP-resistant cells **A.** Two DDP-resistant cells and their parental cells were treated with indicated concentrations of cisplatin for 48 h and then were subjected to MTT assay (n = 5). The results showed that A549/DDP and H1299/DDP cells were more resistant to cisplatin than their parental cells *in vitro*. **B.** Western blotting illustrated increased expression of MDR1 in A549/DDP and H1299/DDP cells. **C.** A549 and H1299 cells displayed epithelial morphology, but A549/DDP and H1299/DDP cells exhibited fibroblastic morphology (original magnification, ×200). **D.** Western blotting showed increased expression of N-cadherin, Vimentin, ZEB1, Snail and reduced expression of E-cadherin in A549/DDP cells. **E.** Wound healing assay and **F.** transwell invasion assay revealed significant enhancement of migration and invasion ability in A549/ DDP cells. Data are means of three separated experiments ± SD, * *P* <0.05, ** *P* <0.01 1. compared with A549 cell group.

Previous studies have demonstrated that chemotherapeutic drug can induce EMT, enhance invasive ability, resulting in drug resistance [31, 32]. Snail and ZEB1 are two crucial EMT inducers [33]. Multi-drug resistance gene 1 (MDR1, ABCB1), encoding P-glycoprotein (P-gp), is one of pharmaceutical carriers that can decrease the effective intracellular concentration of the drug, leading to drug resistance [34]. To determine the mechanism of cisplatin resistance in lung adenocarcinoma cells, we first compared A549/DDP cells and H1299/DDP cells with its parental cells in cisplatin sensitivity, MDR1 expression levels, EMT morphology and related markers expression. MTT assay showed that A549/DDP and H1299/DDP cells exhibited significantly higher resistance to cisplatin than non-DDP-resistant cells (Figure 1A). IC50 of cisplatin in A549/DDP cells was 2.60 fold higher than that in A549 cells and IC50 of cisplatin in H1299/DDP cells was 2.69 fold higher than that in H1299 cells. Western blotting showed that DDP-resistant cells had higher levels of MDR1 protein expression than their non-DDP-resistant cells (Figure 1B). Furthermore, A549 cells and H1299 lung adenocarcinoma cells display epithelial characteristics, whereas A549/DDP and H1299/DDP cells exhibited elongated, fibroblastoid morphology and separated from one another (Figure 1C). Morphological conversion DDP-resistant cells associated with EMT were also reflected by changes in protein levels. Western blotting showed that A549/DDP exhibited downregulation of E-cadherin levels and upregulation of N-cadherin, Vimentin, Snail and ZEB1 (Figure 1D). H1299/DDP cells also have upregulation of N-cadherin, Vimentin, Snail and ZEB1 than H1299 cells, but E-cadherin expression could not be detected (Supplementary Figure 1A). Additionally, wound healing assay and transwell invasion assay demonstrated that the migration and invasion abilities were significantly stronger in A549/DDP cells and H1299/DDP cells (Figure 1E-1F, Supplementary Figure 1B-1C).

### miR-206 overexpression reverses cisplatin resistance, EMT, migration and invasion in DDP-resistant cells

**Figure 2:**
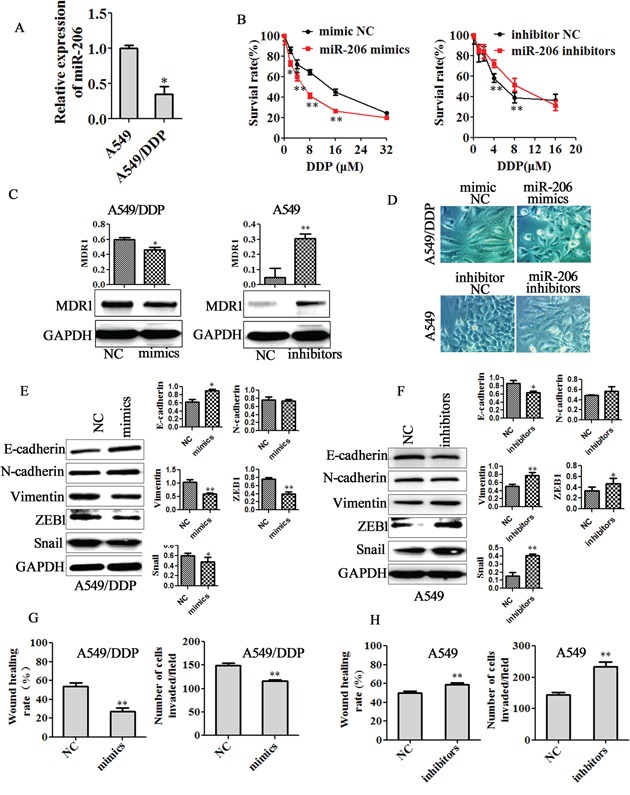
miR-206 decreased cisplatin resistance, EMT, migration and invasion of A549/DDP cells **A.** qRT-PCR assay showed a significant down-regulation of miR-206 in A549/DDP cells compared with in A549 cells. **B.** A549/DDP cells were transfected with miR-206 mimics, and A549 cells were transfected with miR-206 inhibitors. After 24 hrs of transfection, 5×10^3^ cells/well were seeded in 96-well cell culture plates. The next day, cells were incubated with or without the indicated concentration of cisplatin for 48 h and subsequently subjected to an MTT assay. (C-F) A549/DDP cells or A549 cells were transfected with the indicated plasmid. After 48 h, **C.** the expression of MDR1 was determined by Western blotting analysis. **D.** Cell morphology was observed by microscopy (Original magnification, ×200). **E-F.** Western blotting analysis was used to detect the expression of E-cadherin, N-cadherin, Vimentin, ZEB1 and Snail (Left panel), Quantitative results are illustrated for left panel. (G-H) Wound healing assays (Left panel) and invasion assay (Right panel) were used to detect the migration and invasion ability in **G.** miR-206 mimics transfected A549/DDP cells or **H.** miR-206 inhibitors transfected A549 cells. Data are means of three separated experiments ± SD, * *P* <0.05, ** *P* <0.01 compared with their control.

miR-206 has been found to be down-regulated in many types of cancers including lung cancer [21-27, 30]. To determine whether miR-206 plays a pivotal role in drug resistance in lung cancer cells, we measured the expression of miR-206 in the A549/DDP cells, H1299/DDP cells and their parental cells. Real-time PCR assay revealed that miR-206 was significantly lowered in both A549/DDP cells and H1299/DDP cells (Figure 2A, Supplementary Figure 2A) compared with their parental cells. To further validate the role of miR-206 in cisplatin resistance, we transfected miR-206 mimics into A549/DDP cells and H1299/DDP cells, transfected miR-206 inhibitors into A549 cells and H1299 cells. MTT assay revealed that miR-206 mimics treatment led to significantly decreased resistance of A549/DDP cells and H1299/DDP cells to cisplatin, whereas miR-206 inhibitors transfection enhanced the resistance of A549 cells and H1299 cells to cisplatin (Figure 2B, Supplementary Figure 2B-2C). Furthermore, western blotting showed that miR-206 mimics significantly decreased the expression of MDR1 in A549/DDP cells and H1299/DDP cells, while miR-206 inhibitors increased the expression of MDR1 in A549 cells and H1299 cells (Figure 2C, Supplementary Figure 2D).

Previous studies have shown that the drug-resistant cancer cells display features of epithelial-mesenchymal transition (EMT) [32, 35, 36]. Here, we observed that miR-206 mimics transfection led to a change from elongated, fibroblastoid morphology to a rounded shap in both A549/DDP cells and H1299/DDP cells, whereas miR-206 inhibitors transfection resulted in an elongated fibroblast-like morphology of A549 cells and H1299 cells (Figure 2D, Supplementary Figure 2E). Furthermore, miR-206 mimics treatment caused the higher expression of E-cadherin and lower expression of mesenchymal markers including Vimentin, Snail and ZEB1 in A549/DDP cells. Also, miR-206 mimics decreased the expression of N-cadherin, Vimentin, Snail and ZEB1 in H1299/DDP cells (Figure 2E, Supplementary Figure 2F). On the contrary, miR-206 inhibitors reduced E-cadherin expression, induced the expression of Vimentin, ZEB1 and Snail in A549 cells, while induced N-cadherin, Snail and ZEB1expression in H1299 cells (Figure 2F, Supplementary Figure 2G). In addition, invasion and migration assay further demonstrated that miR-206 mimics suppressed the invasion and migration of A549/DDP cells and H1299/DDP cells (Figure 2G, Supplementary Figure 3A-3B), whereas miR-206 inhibitors enhanced the invasion and migration of A549 cells and H1299 cells (Figure 2H, Supplementary Figure 3C-3D). These results indicated that miR-206 could reverses cisplatin resistance, EMT, migration and invasion of cisplatin resistant cells.

### MET gene is a target of miR-206 in lung cancer cells

**Figure 3:**
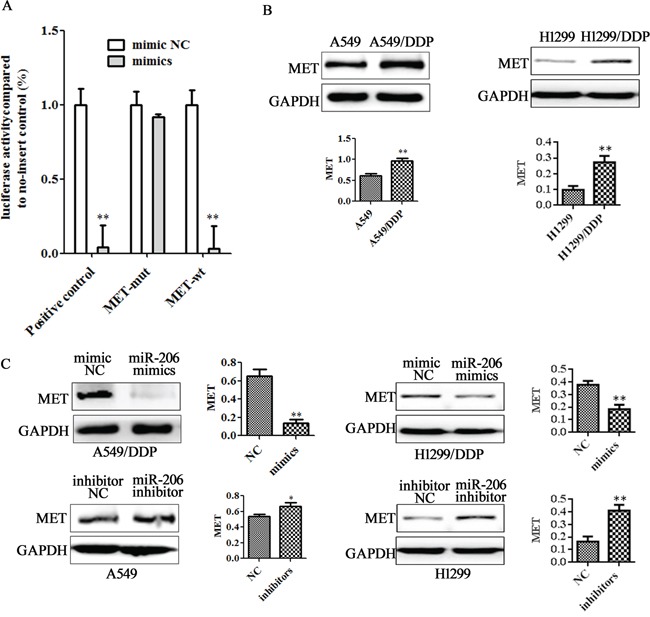
MET is a direct target of miR-206 **A.** Luciferase assay was performed in A549 cells that were cotransfected with miRNA mimics and reporter vectors carrying MET 3'UTR wild type (MET-*wt*), MET 3'UTR mutated type (MET-*mut*), and miR-206 inhibitor sequences (positive control) element. **B.** Variable MET expression in A549 and A549/DDP was obtained by Western blotting analysis. **C.** A549/DDP cells were transfected with miR-206 mimics and A549 cells were transfected with miR-206 inhibotors for 48 h respectively. Western blotting was used to detect MET expression. Data are means of three separated experiments ± SD, ** *P* <0.01, compared with negative control (NC).

Identification of miRNA-regulated gene targets is a necessary step to understand miRNA functions. Based on target prediction programs, we found that MET is a tentative target of miR-206. To test whether the predicted miR-206 target site in the 3′-UTR of MET mRNA was responsible for its regulation, we cloned MET 3′-UTR wild type (MET-wt) or 3′-UTR mutant type (MET-mut) into downstream of the luciferase reporter gene and cotransfected with miR-206 mimics into A549 cells. A luciferase reporter containing miR-206 inhibitor sequence was used as a positive control (PC). As indicated in Figure 3A. Luciferase activity from a construct harboring miR-206 inhibitor sequence (PC group) was significantly decreased in A549 cells expressing either miR-206 or its negative control form. Luciferase activity from A549 cells cotransfected with miR-206 and the construct containing MET-mut form did not induce any significant change in luciferase activity, whereas luciferase activity from A549 cells cotransfected with miR-206 and the construct containing MET-wt was decreased by more than 95% when compared to negative control cells. These results indicate that miR-206 regulates MET protein in A549 cells by directly targeting MET 3′-UTR.

On the other hand, we found that A549/DDP cells and H1299/DDP cells expressed higher levels of MET protein than A549 cells and H1299 cells (Figure 3B), but basal expression of p-MET is undetectable in these cell lines (data not shown). Western blotting showed that miR-206 overexpression could significantly decrease MET expression in A549/DDP and H1299/DDP cells. Meanwhile, down-regulated miR-206 could increase MET expression level in A549 cells and H1299 cells (Figure 3C). These findings further confirmed the existence of an inverse correlation between the expression of miR-206 and MET expression in these cell lines.

### Low expression of miR-206 in lung adenocarcinoma tissues correlates with increased cisplatin resistance and MET expression

**Figure 4:**
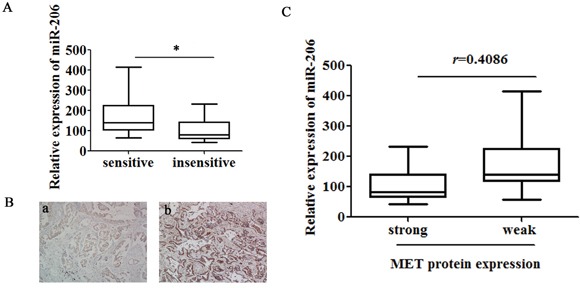
Low expression of miR-206 in lung adenocarcinoma tissues correlates with increased cisplatin resistance and MET expression **A.** Expression levels of miR-206 and **B.** MET protein were detected in cisplatin “sensitive” (a, n =17) and “insensitive” (b, n = 17) lung adenocarcinoma tissues via qRT-PCR (normalized to U6 RNA) and immunostaining (Origninal magnification, ×100), respectively. **C.** The immunoreactivity of MET protein in cisplatin “sensitive” and “insensitive” tissues showed a statistically significant inverse correlation with the relative expression level of miR-206. Data are means of three separated experiments ± SD, * *P* <0.05.

To better understand the association between miR-206 and cisplatin resistance, a total of 34 clinical lung tumor tissue samples were collected from patients with advanced lung adenocarcinoma and divided into “sensitive” and “insensitive” groups according to the patient's response to cisplatin-based chemotherapy. As shown in Figure 4A, miR-206 was significantly down-regulated in the “insensitive” group tissues (n = 17) compared with that in the “sensitive” group (n = 17). Importantly, immunohistochemistry assay showed that 16 of 17 “insensitive” group tissues (94.12%) had positive immunostaining of MET protein but it was 4 of 17 (23.52%) in “sensitive” group (Figure 4B). Moreover, the stronger immunoreactivity of MET was significantly associated with lower miR-206 expression (*r* = 0.4086, *P* = 0.0165, Figure 4C), suggesting that miR-206-MET interaction might be biologically significant in cisplatin resistance. In addition, IHC staining for MET, MDR1, E-cadherin and Vimentin were performed on 5 samples of each group. We found that except Vimentin, all five cisplatin “insensitive” tissues have higher MET and MDR1 expression, and lower E-cadherin expression. In contrast, 3 out of 5 cisplatin “sensitive” tissues have lower MET and MDR1 expression, and higher E-cadherin expression (Supplementary figure 4).

### MET mediated EMT and cisplatin resistance

**Figure 5:**
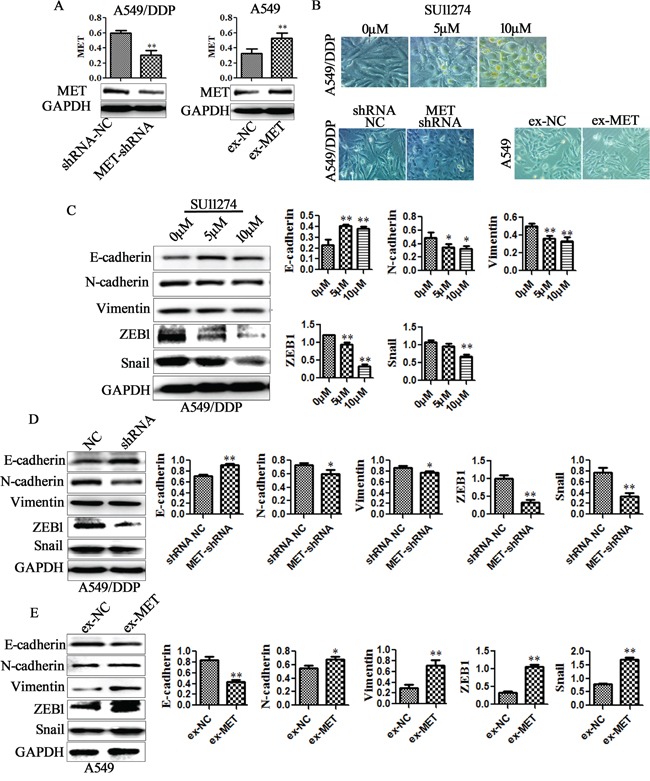
MET is involved in miR-206 inhibited EMT **A.** Western blotting analysis confirmed that the expression of MET was suppressed by MET-shRNA, and activated by MET expression vector (ex-MET). (B-E) A549/DDP cells were treated with MET inhibitor SU11274, or transfected with MET shRNA for 48h, and the A549 cells were transfected with MET expression vector (ex-MET) for 48h, **B.** cell morphological changes associated with EMT are shown in the phase contrast image (Original magnification, ×200). **C-E.** The expression of E-cadherin, N-cadherin, Vimentin, ZEB1, Snail were examined by western blotting. Data are means of three separated experiments ± SD, * *P* <0.05, ** *P* <0.01 compared with their control.

To determine whether the anti-EMT and anti-cisplatin resistance effects of miR-206 on DDP-resistant cells could be partly explained by its targeting of MET, we first analyzed how MET inhibitors, MET silence and MET overexpression affected EMT. Our data showed that the expression of MET was significantly suppressed by MET-shRNA, and activated by MET expression vector (ex-MET) (Figure 5A, Supplementary Figure 5A). MET inhibitor SU11274 treatment partially reversed the mesenchymal phenotype of A549/DDP cells. MET silence also reversed the mesenchymal phenotype in both A549/DDP cells and H1299/DDP cells, while MET overexpression induced a mesenchymal phenotype in A549 cells and H1299 cells (Figure 5B, Supplementary Figure 5B). Western blotting analysis showed that both MET inhibitor SU11274 and MET-shRNA increased the E-cadherin protein expression, but decreased the expression of mesenchymal markers including N-cadherin, Vimentin, ZEB1 and Snail (Figure 5C-5D, Supplementary Figure 5D). In contrast, MET overexpression decreased the E-cadherin protein expression, but increased the expression of N-cadherin, Vimentin, ZEB1 and Snail in A549 cells (Figure 5E). Similarly, MET-shRNA decreased the expression of N-cadherin, Vimentin, ZEB1 and Snail (Supplementary Figure 5B). In contrast, MET overexpression increased the expression of N-cadherin, Vimentin, ZEB1 and Snail in A549 cells (Supplementary Figure 5C). However, E-cadherin expression could not be detected in both conditions.

**Figure 6:**
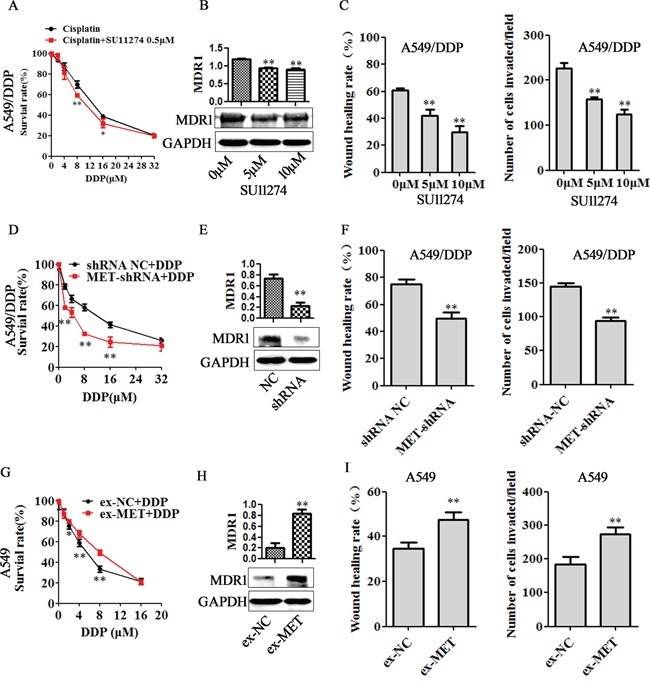
MET is involved in miR-206 inhibited cisplatin resistance A549/DDP cells were treated with MET inhibitor SU11274 (indicated concentration), or were transfected with MET shRNA for 48h, and A549 cells were transfected with MET expression vector (ex-MET) for 48h, **A, D** and **G**. the changes of cisplatin sensitivity, **B, E** and **H.** MDR1 expression, **C, F** and **I**. migration and invasion ability in each groups were detected. Data are means of three separated experiments ± SD, * *P* <0.05, ** *P* <0.01 compared with their control.

We next analyzed the effect of MET inhibitors, MET silence and MET overexpression on cell cisplatin sensitivity, migratory and invasive capability. As shown in Figure 6, SU11274 significantly promoted cell growth inhibition induced by cisplatin (Figure 6A) and decreased the MDR1 expression in A549/DDP cells (Figure 6B). Similarly, MET shNA transfection also increased the sensitivity of A549/DDP cells to cisplatin (Figure 6D) and decreased the MDR1 expression (Figure 6E). IC50 in SU11274 (0.5μM) group and MET shRNA group were 10.58μM and 3.53μM, significantly lower than their DDP control groups (12.82μM and 10.06μM, respectively. Supplementary Table 1). Furthermore, SU11274 treatment and MET shRNA transfection showed similar effects on suppressing migration and invasion of A549/DDP cells (Figure 6C and Figure 6F). In contrast, MET overexpression increased the cisplatin resistance and MDR1 protein expression (Figure 6G-6H, Supplementary Table 1), enhanced the capability of cell migration and invasion (Figure 6I). In H1299/DDP cells, MET silence decreased the MDR1 expression (Supplementary Figure 5E), suppressed cell migration and invasion (Supplementary Figure 6A-6B). While MET overexpression increased MDR1 protein expression (Supplementary Figure 5F), enhanced the capability of H1299 cell migration and invasion (Supplementary Figure 6C-6D).

### miR-206 inhibits EMT and cisplatin resistance via MET dependent PI3K/AKT /mTOR signaling pathways

**Figure 7:**
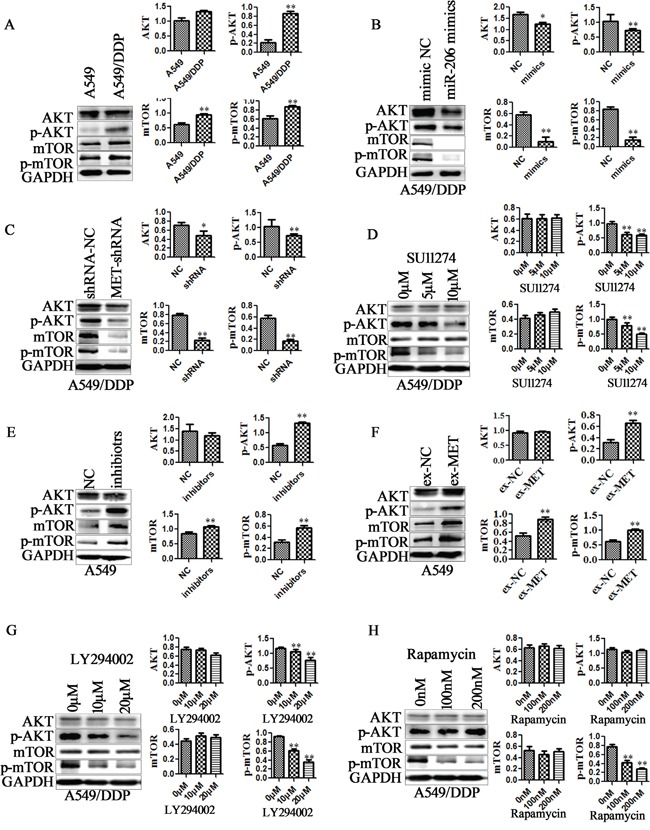
miR-206/MET regulated PI3K/AKT/mTOR pathway Western blotting analysis was performed to detect the protein expression of AKT, p-AKT, mTOR, p-mTOR in **A.** A549/DDP cells and A549 cells, **B.** miR-206 mimics transfected A549/DDP cells, **C.** MET shRNA transfected A549/DDP cells, **D.** MET inhibitors SU11274 treated A549/DDP cells, **E.** miR-206 inhibitors transfected A549 cells, **F.** MET overexpression vectors (ex-MET) transfected A549 cells, **G.** PI3K inhibitor LY294002 treated A549/DDP cells, **H.** mTOR inhibitor rapamycin treated A549/DDP cells. Cell lysates were collected 48 h after transfection or 2h after treatment with MET, PI3K and mTOR inhibitors. Data are means of three separated experiments ± SD, * *P* <0.05, ** *P* <0.01 compared with their control.

PI3K/AKT/mTOR is one of the definite downstream targets of MET receptor. Inhibition of PI3K/AKT signaling has proven to be an efficient way to attenuate the resistance of chemotherapy [37]. In present study, we found that AKT/mTOR pathway is activated in A549/DDP cells and H1299/DDP cells compared with the parental cells (Figure 7A, Supplementary Figure 7A). To determine whether miR-206 inhibits MET dependent PI3K/AKT/mTOR signaling pathways, we analyzed the effects of miR-206 or MET expression changes on PI3K/AKT/mTOR signaling. The results showed that both miR-206 mimics transfection and MET-shRNA treatment significantly decreased AKT, p-AKT, mTOR and p-mTOR protein levels in A549/DDP cells (Figure 7B-7C), and MET inhibitor SU11274 also resulted in a decrease in p-AKT, p-mTOR protein expression(Figure 7D). In contrast, miR-206 inhibitors and MET overexpression increased p-AKT, mTOR and p-mTOR protein levels in A549 cells (Figure 7E-7F). Although the degree of gene expression and protein levels varied between different cell lines, similar results were also found in H1299/DDP and H1299 cell lines (Supplementary Figure 7B-7C).

**Figure 8:**
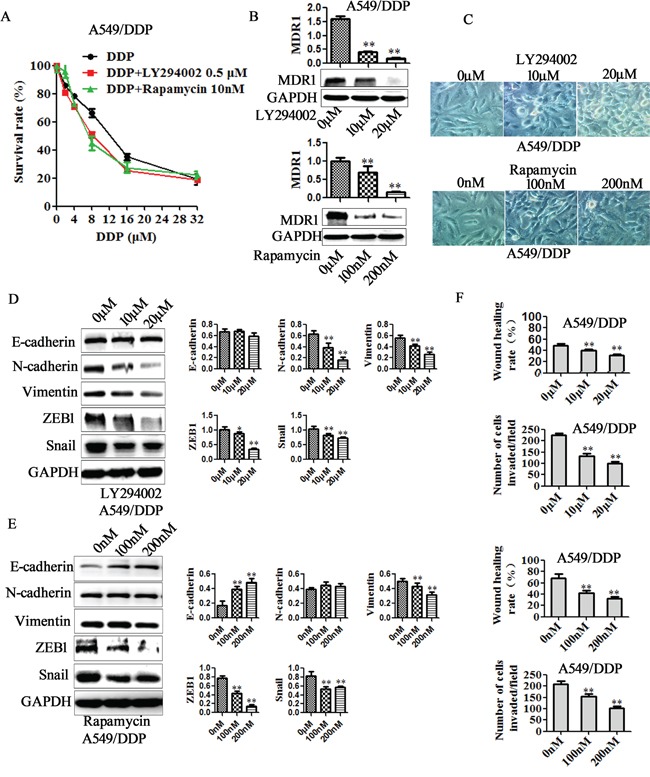
PI3K/AKT/mTOR pathway is involved in miR-206/MET regulated cisplatin resistance, EMT, migration and invasion A549/DDP cells were treated with LY294002 (indicated concentration) or rapamycin (indicated concentration) respectively. After 48h, **A.** The changes of cisplatin sensitivity, **B.** MDR1 expression, **C.** cell morphology (Original magnification, ×200), **D-E.** E-cadherin, N-cadherin, Vimentin, ZEB1 and Snail expression, **F.** migration and invasion ability in each group of cells were detected. Data are means of three separated experiments ± SD, * *P* <0.05, ** *P* <0.01 compared with their control.

To further support the role of PI3K/AKT/mTOR signaling in suppression of EMT and cisplatin resistance by miR-206, PI3K selective inhibitor LY294002 and mTOR inhibitor rapamycin was utilized in A549/DDP cells. LY294002 (10μM, 20μM) were observed to remarkably reduce the protein of p-AKT and p-mTOR, and rapamycin to reduce p-mTOR in A549/DDP cells (Figure 7G-7H). Furthermore, both LY294002 (0.5μM) and rapamycin (10nM) enhanced cisplatin sensitivity, decreased the MDR1 expression in A549/DDP cells (Figure 8A–8B). IC50 decreased from 11.12 μM to 7.78 μM for LY294002, and decreased from 11.12 μM to 8.31 μM for rapamycin (Supplimentary Table 1). In addition, both LY294002 and rapamycin reversed mesenchymal characteristics (Figure 8C), decreased the expression of mesenchymal markers (Figure 8D-8E), and inhibited the migration and invasion of A549/DDP cells (Figure 8F). These data indicated the involvement PI3K/AKT/mTOR pathway in suppression of EMT and cisplatin resistance by miR-206.

### miR-206 enhances A549/DDP cells to cisplatin sensitivity *in vivo*

**Figure 9:**
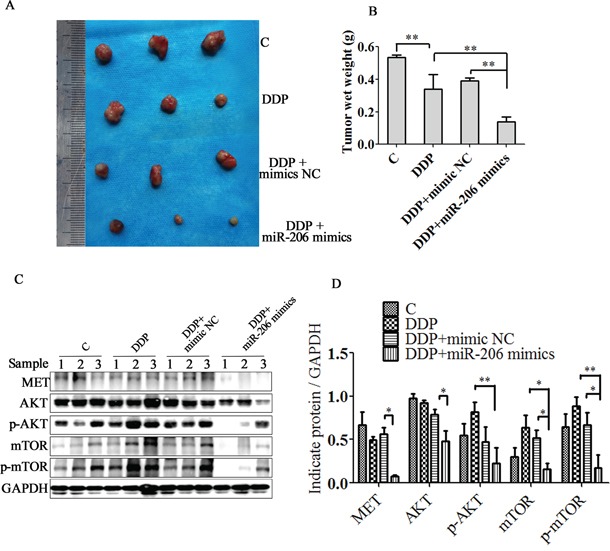
miR-206 enhances A549/DDP cells to cisplatin sensitivity *in vivo* **A.** The gross morphology of tumor samples **B.** The final xenograft tumor weights were measured after 30 days of treatment. **C.** MET/AKT/ mTOR pathway proteins expression in excised xenograft tumor were detected by western blotting assay. **D.** Relatve expression levels of MET/AKT/mTOR pathway proteins. sample1, 2, 3 stand for mouse tumor tissues from each group. Data are means of three separated experiments ± SD, (* *P* < 0.05, ** *P* < 0.01 compared with indicated control).

To further investigate the effect of miR-206 expression on cisplatin sensitivity, we evaluated the *in vivo* antitumor activity of miR-206 in xenograft model. As shown in Figure 9A-9B, The *in vivo* results parallel the *in vitro* results and show that miR-206 resulted in dramatic tumor regressions compared with both negative control or DDP control group. Furthermore, the expression of MET, AKT, p-AKT, mTOR and p-mTOR were significantly decreased in the miRNA-206 plus cisplatin group compared with DDP combined mimic NC group or DDP group. (Figure 9C-9D). These results further suggest that miR-206 and its downstream MET/AKT/mTOR pathway play important roles in controlling A549/DDP cells cisplatin sensitivity.

## DISCUSSION

Previous studies have reported that miR-206 could act as a tumor-suppressor in various cancers including lung cancer [21–30]. We and others have demonstrated that miR-206 overexpression inhibited invasion and metastasis in lung cancer cells [26, 30]. In the current study, we demonstrated that miR-206 could suppress EMT process and cisplatin resistance of lung adenocarcinoma cells, partly through targeting MET and its downstream PI3K/AKT/ mTOR pathway both *in vitro* and *in vivo*. Our data provide a first insight into the function of miR-206 in regulating cisplatin resistance and EMT in cisplatin resistant lung adenocarcinoma cells.

The EMT is a molecular process through which an epithelial cell undergoes transdifferentiation into a mesenchymal phenotype. Recent evidences have suggested that EMT processes may play an important role in the development of chemoresistance. It has been reported knockdown of snail and slug reverses the EMT phenotype and reduces ovarian cancer cell resistance to cisplatin [17]. Inhibiting EMT by overexpressing the microRNA miR-200 could abrogate cyclophosphamide resistance in spontaneous breast-to-lung metastasis models [38]. Furthermore, in pancreatic ductal adenocarcinoma (PDAC) mouse models, EMT suppression by Snail or Twist deletion leads to an increase in cancer cell proliferation with enhanced expression of nucleoside transporters in tumours, contributing to enhanced sensitivity to gemcitabine treatment and increased overall survival of mice [39]. In our models of cisplatin resistant lung cancers, we found that EMT gene signatures were also significantly correlated with the presence of cisplatin resistance and increasing expression of MDR1. The results further suggested a close correlation between EMT and cisplatin resistance in lung cancer cells.

It is becoming increasingly evident that miRNAs are key modulators of cisplatin resistance and EMT in lung cancer. For instance, miR-451 is downregulated in NSCLC tissues and is capable of conferring resistance to cisplatin in non-small cell lung cancer cell line (A549) [40]. miR-224 can promote both *in vitro* and *in vivo* cisplatin resistance of A549 cells via targeting gene p21 (WAF1/CIP1) and regulating G1/S cell cycle transition and apoptosis [16]. Moreover, it has been reported that downregulation of miRNA-27a is responsible for EMT and cisplatin resistance in A549 cells by directly targeting Raf Kinase Inhibitory Protein (RKIP) [41]. In support of the role of miRNAs in cisplatin resistance and EMT in lung cancer, our study identified that miR-206 is down-regulated and could confer cisplatin resistance and EMT in A549/DDP cells and H1299/DDP cells.

Targeting MET might be an effective way to enhance cisplatin sensitivity in certain tumors. It has been reported that MET inhibition in osteosarcoma cancer is associated with higher tumor aggressive behavior and resistance to cisplatin therapy [42], and overexpression of MET enhances survival of ovarian cancer cells and increased resistance to cisplatin [43]. Moreover, HGF increases cisplatin resistance via activation of MET in lung cancer cells [27]. Consistent with these reports, our data showed that downregulation of MET reduced EMT process and reversed cisplatin resistance in A549/DDP cells and H1299/DDP cells. Interestingly, we found that MET is also one of the targets of miR-206. Despite MET has recently been identified as a target of miR-206 in rhabdomyosarcoma cells [44]. We further demonstrated that miR-206 directly target MET in lung cancer A549 cells. In addition, our results in clinical lung cancer tissue samples show that the decreased expression of miR-206 closely correlated with increased MET expression and poor cisplatin sensitivity. These data suggest that miR-206-MET play important roles in regulating EMT and cisplatin resistance in lung adenocarcinoma cells.

PI3K/AKT/mTOR is a crucial downstream pathway of MET and can regulate many of the biological phenomena, such as cell proliferation and survival, motility and migration, and tumor cell invasion. In the present study, we found miR-206-MET axis regualted PI3K/AKT/mTOR pathway in lung cancer cells. Both miR-206 mimics and MET-shRNA suppresses the phosphorylation of AKT/mTOR in A549/DDP cells. In contrast, miR-206 inhibitors or MET overexpression enhanced phosphorylation of AKT/ mTOR in A549 cells. To further investigate the molecular mechanisms linking the PI3K/AKT/mTOR pathway and DDP resistance, we detected the expression of MDR1, a multispecific efflux transporter of drugs, after PI3K selective inhibitor LY294002 or mTOR inhibitor rapamycin treatmnet in A549/DDP cells. The results showed that both these two inhibitors reduced the expression of MDR1. In contrast, miR-206 inhibitors and MET overexpression activated the PI3K/AKT/mTOR pathway and increased MDR1 expression in A549 cells. These results provides a possible mechanism linking miR-206, MET/PI3K/AKT/ mTOR pathway, cisplatin resistance, by which downregulated expression of multi-drug resistance genes leads to cisplatin resistance in A549 cells. In addition, We further detected the expression of two transcription factors ZEB1 and Snail, which are two crucial EMT activators. We demonstrated that decrease of EMT related transcription factors, such as ZEB1 and Snail expression is one of the molecular mechanisms from the deregulated miR-206 levels to the EMT.

In the present study, we also assessed the anti-tumour effect of miR-206 in a cisplatin-resistant *in vivo* mice model. We found that miR-206 inhibited the MET/AKT/ mTOR pathway and enhanced the A549/DDP cell sensitivity to cisplatin *in vivo*. Therefore, these results further demonstrated *in vivo* that miR-206 inhibiting MET and its downstream PI3K/AKT/mTOR pathways is one potential mechanism to overcome cisplatin resistance in lung cancer. To the best of our knowledge, we provided a first insight into the roles and possible mechanisms of miR-206 upregulation in chemosensitivity of A549 cells to cisplatin. However, because only two pairs of cisplatin-resistance cell lines were used in our study, further investigation in other lung adenocarcinoma cell lines is necessary to explore the function and mechanisms of miR-206 in cisplatin resistance.

Taken together, our study demonstrated that miR-206 overexpression in human lung adenocarcinoma cisplatin resistant cells inhibited the EMT and cisplatin resistance by targeting MET and suppressing its downstream PI3K/AKT/mTOR signaling pathway. Low expression of miR-206 and high levels of MET were strongly associated with the poor cisplatin sensitivity of lung adenocarcinoma patients. Therefore, activation of miR-206 or inactivation of its target gene pathway may be a potential strategy to reverse cisplatin resistance in human lung adenocarcinoma cisplatin resistant cells.

## MATERIALS AND METHODS

### Cell culture

Human lung cancer cell line A549, H1299 and A549/DDP were obtained from China Center for Type Culture Collection (CCTCC, Shanghai, China). All the cells were cultured in RPMI-1640 medium supplemented with 10% fetal bovine serum (FBS, Gibco, USA) in a humidified atmosphere containing 5% CO_2_ at 37¼C. To establish cisplatin-resistant H1299 cell lines, H1299 cells were first treated with 0.6 μM of cisplatin (DDP, Sigma, St. Louis, MO), and then were treated with increased concentrations of DDP in a stepwise manner during each passage. To maintain the drug-resistant phenotype, DDP (with final concentration of 2μM) was added to the culture media for A549/DDP cells and H1299/DDP cells.

### Reagents and antibodies

MET inhibitor (SU11274) and PI3 kinase inhibitor (LY294002) were purchased from Selleck Chemicals (Houston, TX). Primary antibodies phospho-MET (Y1234/35), phospho-AKT (S473), phospho-mTOR (S2448), MET, AKT, mTOR, E-cadherin, N-cadherin, Vimentin, ZEB1, Snail and GAPDH were purchased from Cell Signaling Technology (Beverley, MA). MDR1 antibody was purchased from Santa Cruz Biotechnology (Santa Cruz, CA). Secondary antibodies, HRP-conjugated goat anti-mouse IgG and goat anti-rabbit IgG, were obtained from Jackson (West Grove, PA).

### Tissue samples

A total of 34 lung adenocarcinoma tissues were collected from patients with advanced lung adenocarcinoma who received chemotherapy at The 117th Hospital of PLA (Hangzhou, China) between June 2013 and June 2014. Informed consent was obtained from all subjects and thisstudy was approved by the Clinical Research Ethics Committee of The 117th Hospital of PLA. Patients met all of the following criteria: primary lung adenocarcinoma; histological diagnosis of lung adenocarcinoma with at least 1 measurable lesion; clinical stage IIIB-IV; first-line chemotherapy either with cisplatin 100 mg/m^2^ and pemetrexed 500 mg/m^2^ or cisplatin 100 mg/m^2^ and gemcitabine 1000 mg/m^2^ administered every 3 weeks for a maximum of 5 cycles. Samples were divided into “sensitive” (complete response or partial response) and “insensitive” (stable disease or progressive disease) groups according to the patient's responses assessed via medical image analysis and detection of serum tumor markers after 4 or 5 cycles of cisplatin-based chemotherapy.

### Transient transfection

FAM-labled mimic negative control (mimic NC), miR-206 mimics (mimics), inhibitor NC, miR-206 inhibitors, MET silence vectors p-GPU6-MET-shRNA (MET-shRNA), shRNA control, MET (Accession NO: NM_000245) overexpression vector pEZ/M98/neo-MET (ex-MET) and the ex-control were purchased from GenePharma (Shanghai, China). The MET shRNA sequences were designed as showed in Supplimentary Table 2. The cells were seeded into 6-well plates and transfected with 75 pmol oligonucleotides or 2.5μg shRNA vectors using Lipofectamine 2000 (Invitrogen, USA) according to the instructions provided by the manufacturer. The cells were used for further analysis 48h after transfection.

### Quantitative real-time PCR analysis

Total RNA was isolated with Trizol reagent (Invitrogen, USA). The concentration and purity of the RNA samples were determined spectroscopically. Expression of mature miRNA was assayed using stem-loop RT followed by real-time PCR analysis. The SYBR and U6 gene were used for detecting the gene amplification and normalizing the each sample, respectively. The primers for RT-PCR were designed based on the miR-206 sequences provided by the Sanger Center miRNA Registry. The RT primers were designed as showed in Supplimentary Table 3. qRT-PCR was performed on the ABI (Applied Biosystems) 7900 HT Thermal cycler in standard mode for 40 cycles. The fold change was calculated using the 2 ^−ΔΔCt^ Method. PCR was performed in triplicate.

### Luciferase reporter assay

Based on the miRNA databases (microRNA.org, miRDB and TargetScan database), MET is a predicted target of miR-206 in humans. According to the results of prediction, we cloned MET 3'UTR fragment containing the predicted site into pGL3 luciferase reporter vector (pmirGLO3, Promega, Madison, USA) and named as MET-wt. We cloned MET 3'UTR fragment with mutant sequence into pmirGLO3 luciferase reporter vector and named as MET-mut. In addition, miR-206 inhibitor sequence was also cloned into pmirGLO3 luciferase reporter vector as a positive control (PC). For luciferase assay, the reporter plasmid was cotransfected with miR-206 mimics or mimic NC in A549 cells. After 48 h, cells were harvested, and the luciferase activity was measured using the Dual-Luciferase Reporter Assay System (Promega, Madison, USA).

### Migration and invasion assay

Wound healing experiment and Transwell insert (24-well insert; pore size 8μm, Corning, USA) assays were used to determine the migration and invasion abilities of the cells, respectively. Briefly, for the wound healing experiment, cells were grown to confuence wounded using a pipette tip and photographed at 0 h and subsequent time points. Cell migration was evaluated by measuring the width of the wound at the identical position. For the invasion assay, the lower chambers of matrigel-coated invasion plates were used. Cells (50,000) were added to the upper chamber in serum-free media and invasion at 37¼C towards 10% FBS-containing growth media was determined after 24 h. Cells that invaded through the membrane were fixed, stained with crystal violet and photographed. The invaded cells were counted by Image J software. All experiments were carried out in triplicate.

### *In vitro* drug sensitivity assay

The cells were plated in 6-well plates (3×10^5^ cells/well) and 75 pmol of the miR-206 mimic or negative control were transfected into the A549/DDP cells, while a miR-206 inhibitors or inhibitor negative control were transfected into the A549 cells, using Lipofectamine-2000 (Invitrogen, USA) according to the manufacturer's instructions. Twenty-four hours after transfection, the cells were seeded in 96-well plates (5×10^3^cells/well) for the following experiment. After cell adhesion, freshly prepared anticancer drug (cisplatin; Qilu Pharmaceutical Co., Ltd., Jinan, China) was added at a final concentration of 2, 4, 8, 16, 32 μM for A549/DDP, and 1, 2, 4, 8, 16 μM for A549 cells. Forty-eight hours after the addition of the drug, cell viability was assessed by the 3-(4,5-dimethylthiazol-2-yl) -2,5-diphenyl-tetrazolium bromide (MTT) assay. The absorbance at 490 nm (A490) of each well was read on a spectrophotometer. The concentration at which the drug produced 50% inhibition of growth (IC50) was estimated by the relative survival curve. Three independent experiments were performed in duplicate.

### Western blot analysis

Cells were washed in PBS and lysed in RIPA lysis buffer supplemented with protease inhibitor cocktail (Roche, Germany). Total protein was quantfied by BCA Protein Assay Kit (Beyotime, Nanjing, China), and an equal amount of whole cell lysates was resolved by SDS-polyacrylamide gel electrophoresis (PAGE) and transferred to a polyvinylidene dfluoride (PVDF) membrane (Millipore, Germany). The blots were blocked in BSA (5% w/v in PBS + 0.1% Tween 20) for 1 h at room temperature and immunostained with antibodies at 4¼C overnight. Immunoreactive bands were visualized by enhanced chemiluminescence (Millipore, Germany) according to the manufacturer's instructions. Data were normalized to GAPDH.

### Immunohistochemistry

Tissue slides were incubated for 2 h at 56¼C and de-paraffinized. Antigen retrieval was obtained by microwave treatment in citrate buffer for 15 min to retrieve antigenicity. After peroxidase activity was blocked with 3% H_2_O_2_/methanol for 10 min, sections were incubated with normal goat serum for 20 min to block non-specific antibody binding sites. Sections were incubated with the primary antibodies for 1 h at 25°C followed by incubations with biotinylated anti-rabbit/mouse IgG and peroxidase-labelled streptavidin for 10 min each. The percentage of the cells with cytoplasmic labeling was recorded from two areas of each specimen, and the labeling intensity was estimated as 1+, 2+ or 3+. The immunohistochemistry results were categorized into two groups: the samples without any labeling, 1+ labeling in <25% cells, and 2+ labeling in <5% cells were considered negative; all the remaining samples were defined as positive.

### Animal studies

All experimental procedures used in this study had been approved by the ethics committee in the 117th Hospital of PLA. Male nude mice (BALB/c, 4-6wk) were purchased from Shanghai Laboratory Animal Center (Shanghai, China). For preparation of subcutaneous xenograft model, 0.2 ml A549/DDP lung cancer cells (2.0 × 10^6^) in phosphate buffered saline/100 μl were injected subcutaneously into the right flank of the nude mice. 15 days after cell inoculation, total of 20 mice were divided randomly into four groups (five mice per group). miR-206 agomirs and miR-206 agomir negative control (NC) (2 nmol; Genepharma, Shanghai, China) were given locally by direct injection into the xenografts every two days. Meanwhile, cisplatin was administered via intraperitoneal injection at a dose of 5 mg/kg every other day. After 30 days of treatment, all mice were sacrificed. Transplanted tumors were excised, and the wet weight was recorded. Protein expression in tumors was detected by western blotting assay.

### Statistical analysis

All statistical analyses were performed using SPSS 13.0. Numerical data were presented as mean ± SD. The statistical difference of data between groups was analyzed by one-way analysis of variance (ANOVA) and Student's t test. Differences were considered significant when *P* < 0.05.

## SUPPLEMENTARY FIGURES AND TABLES


