# Supplementary material for: miR-206 regulates cisplatin resistance and EMT in human lung adenocarcinoma cells partly by targeting MET
Source: Oncotarget. 2016 Mar 21;7(17):24510–26. doi: 10.18632/oncotarget.8229 (PMC5029718; doi:10.18632/oncotarget.8229)
Supplement: Supplementary file 1 [file oncotarget-07-24510-s001.pdf]

SUPPLEMENTARY FIGURES AND TABLES

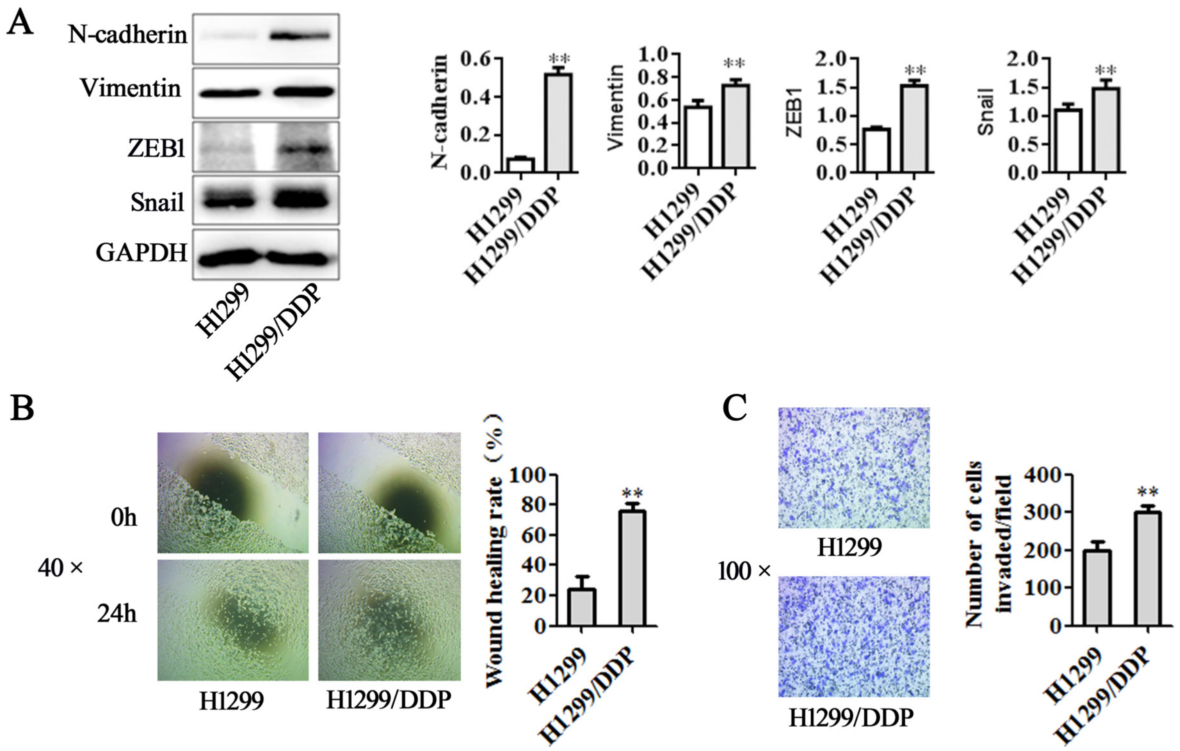

Supplementary Figure 1: Differences between H1299/DDP cells and parental H1299 cells. A. Western blotting showed increased expression of N-cadherin, Vimentin, ZEB1, Snail in H1299/DDP cells, the expression of E-cadherin was undetectable in both H1299/DDP and H1299 cells. **B.** Wound healing assay and **C.** transwell invasion assay revealed significant enhancement of migration and invasion ability in H1299/DDP cells. Data are means of three separated experiments ± SD, * P <0.05, ** P <0.01 compared with their contorl.

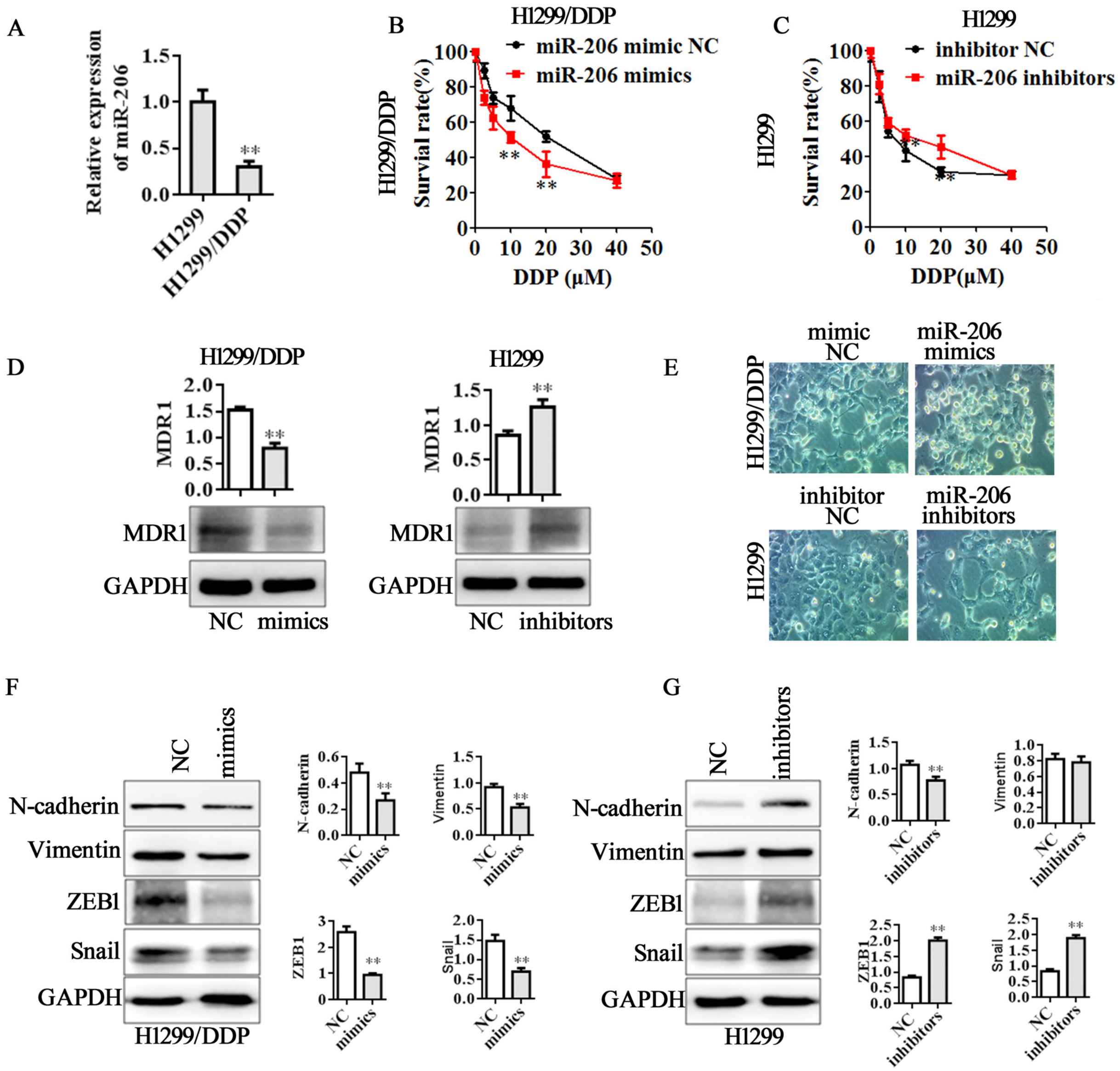

Supplementary Figure 2: miR-206 decreased cisplatin resistance, EMT of H1299/DDP cells. **A.** qRT-PCR assay showed a significant down-regulation of miR-206 in H1299/DDP cells compared with in H1299 cells. **B-C.** H1299/DDP cells were transfected with miR-206 mimics, and H1299 cells were transfected with miR-206 inhibitors. After 24 hrs of transfection, 5×10^3 cells/well were seeded in 96-well cell culture plates. The next day, cells were incubated with or without the indicated concentration of cisplatin for 48 h and subsequently subjected to an MTT assay. **D.** H1299/DDP cells and H1299 cells were transfected with the miR-206 mimics and inhibitors respectively. After 48 h, the expression of MDR1 was determined by Western blotting analysis. **E.** Cell morphology was observed by microscopy (Original magnification, ×200). **F-G.** Western blotting analysis was used to detect the expression of N-cadherin, Vimentin, ZEB1 and Snail (Left panel), Quantitative results are illustrated (Right panel). Data are means of three separated experiments ± SD, * P <0.05, ** P <0.01 compared with their control.

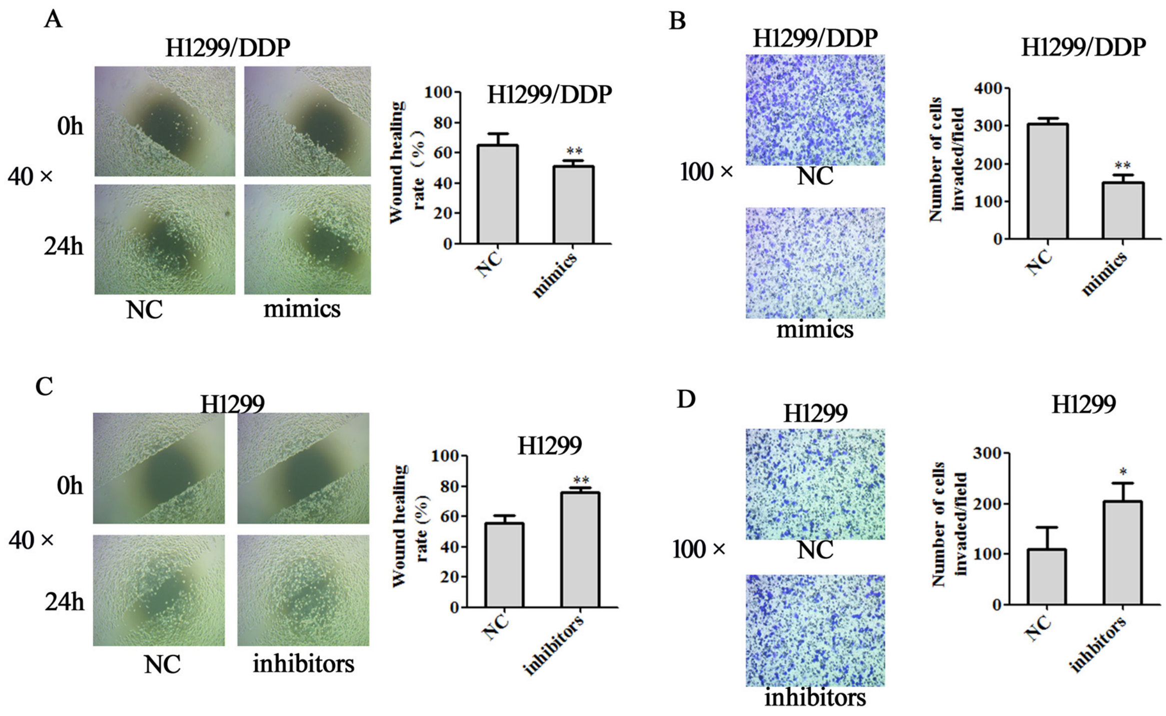

Supplementary Figure 3: miR-206 decreased migration and invasion of H1299/DDP cells. H1299/DDP cells were transfected with miR-206 mimics, and H1299 cells were transfected with miR-206 inhibitors. Wound healing assays **A-C.** and invasion assay **B-D.** were used to detect the migration and invasion ability in miR-206 mimics transfected H1299/DDP cells or miR-206 inhibitors transfected H1299 cells. Data are means of three separated experiments ± SD, * P <0.05, ** P <0.01 compared with their control.

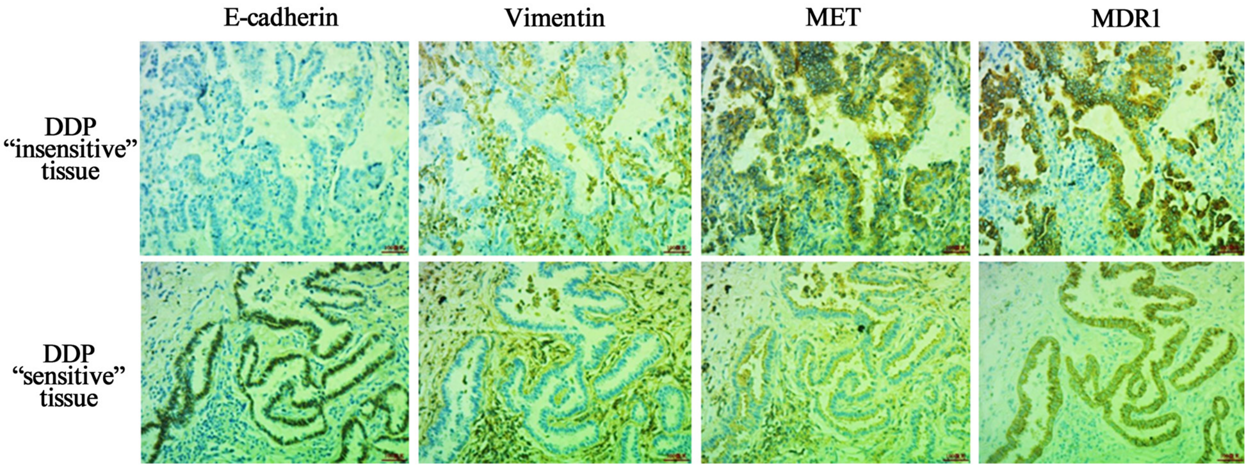

Supplementary Figure 4: Correlation of the E-cadherin, Vimentin, MET and MDR1 protein expression in DDP sensitive and insensitive tissues. Expression levels of E-cadherin, Vimentin, MET and MDR1 were detected in cisplatin "sensitive" (n =5) and "insensitive" (n = 5) lung adenocarcinoma tissues via immunohistochemistry method. The expression of E-cadherin was decreased, while the expression of MET and MDR1were increased in the insensitive tissues, but the expression of Vimentin protein has not show significant difference compared with sensitive tissues.

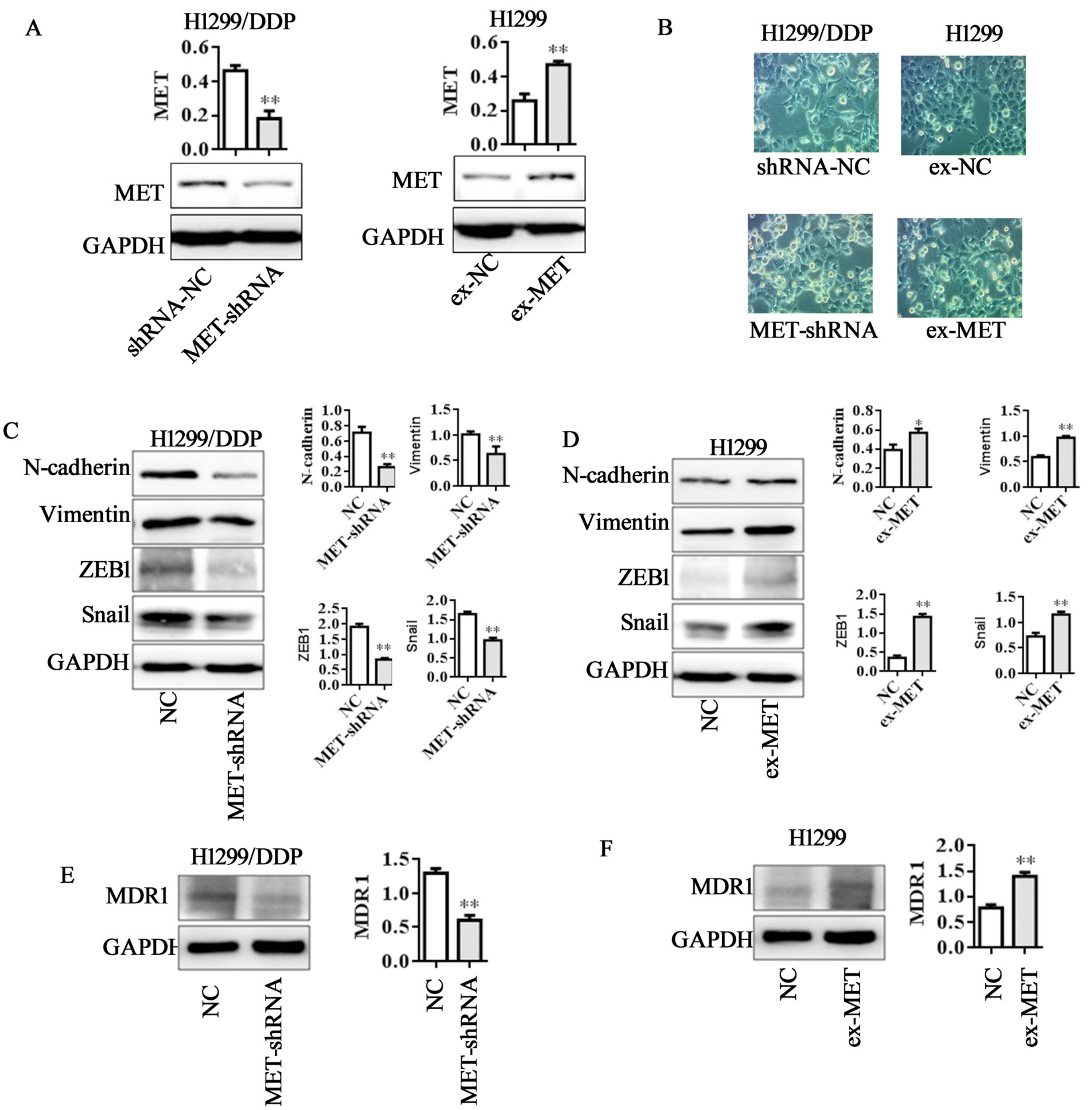

Supplementary Figure 5: MET is involved in miR-206 inhibited EMT and cisplatin resistance in H1299/DDP cells. **A.** H1299/DDP cells were transfected with MET shRNA for 48h, and the H1299 cells were transfected with MET expression vector (ex-MET) for 48h, Western blotting analysis confirmed that the expression of MET was suppressed by MET-shRNA, and activated by MET expression vector (ex-MET). **B.** Cell morphological changes associated with EMT are shown in the phase contrast image (Original magnification, ×200). **C-F.** The expression of N-cadherin, Vimentin, ZEB1, Snail, MDR1 were examined by western blotting. Data are means of three separated experiments ± SD, * P <0.05, ** P <0.01 compared with their control.

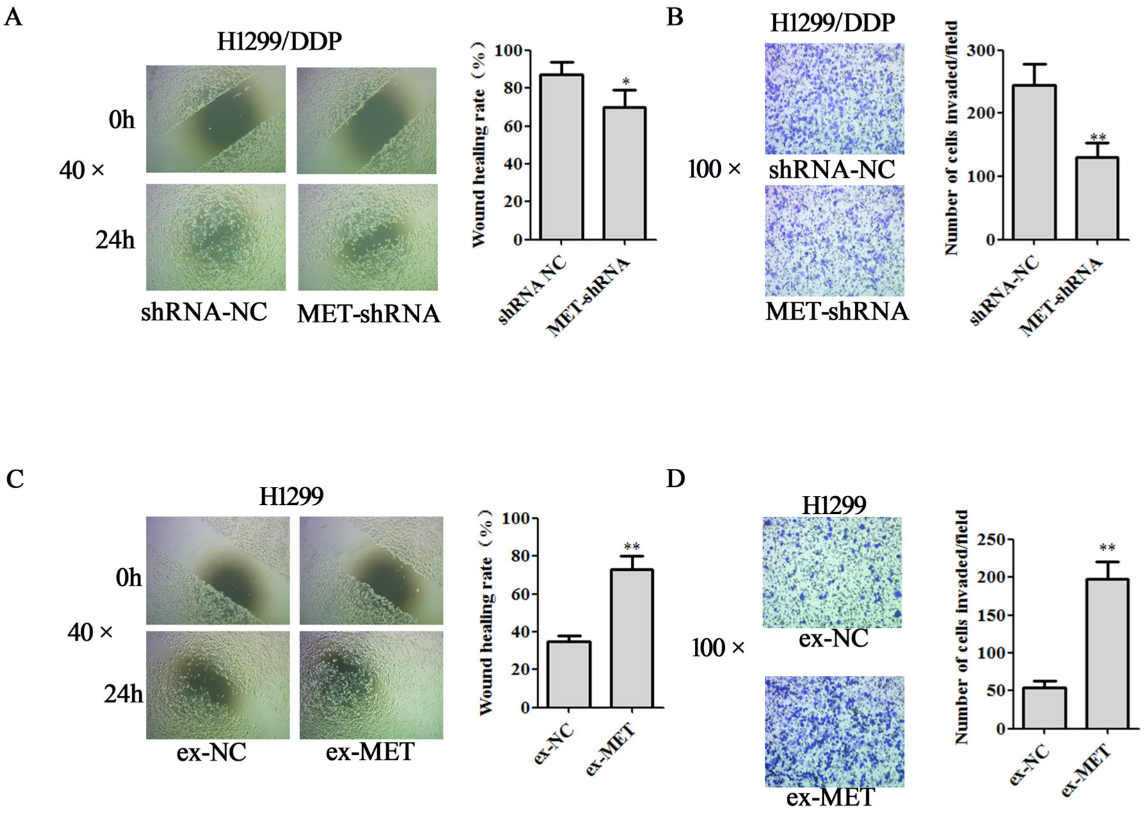

Supplementary Figure 6: MET is involved in miR-206-decreased migration and invasion of H1299/DDP cells. H1299/DDP cells were transfected with MET shRNA for 48h, and H1299 cells were transfected with MET expression vector (ex-MET) for 48h, Wound healing assays **A-C.** and invasion assay **B-D.** were used to detect the migration and invasion ability in transfected cells. Data are means of three separated experiments ± SD, * P <0.05, ** P <0.01 compared with their control.

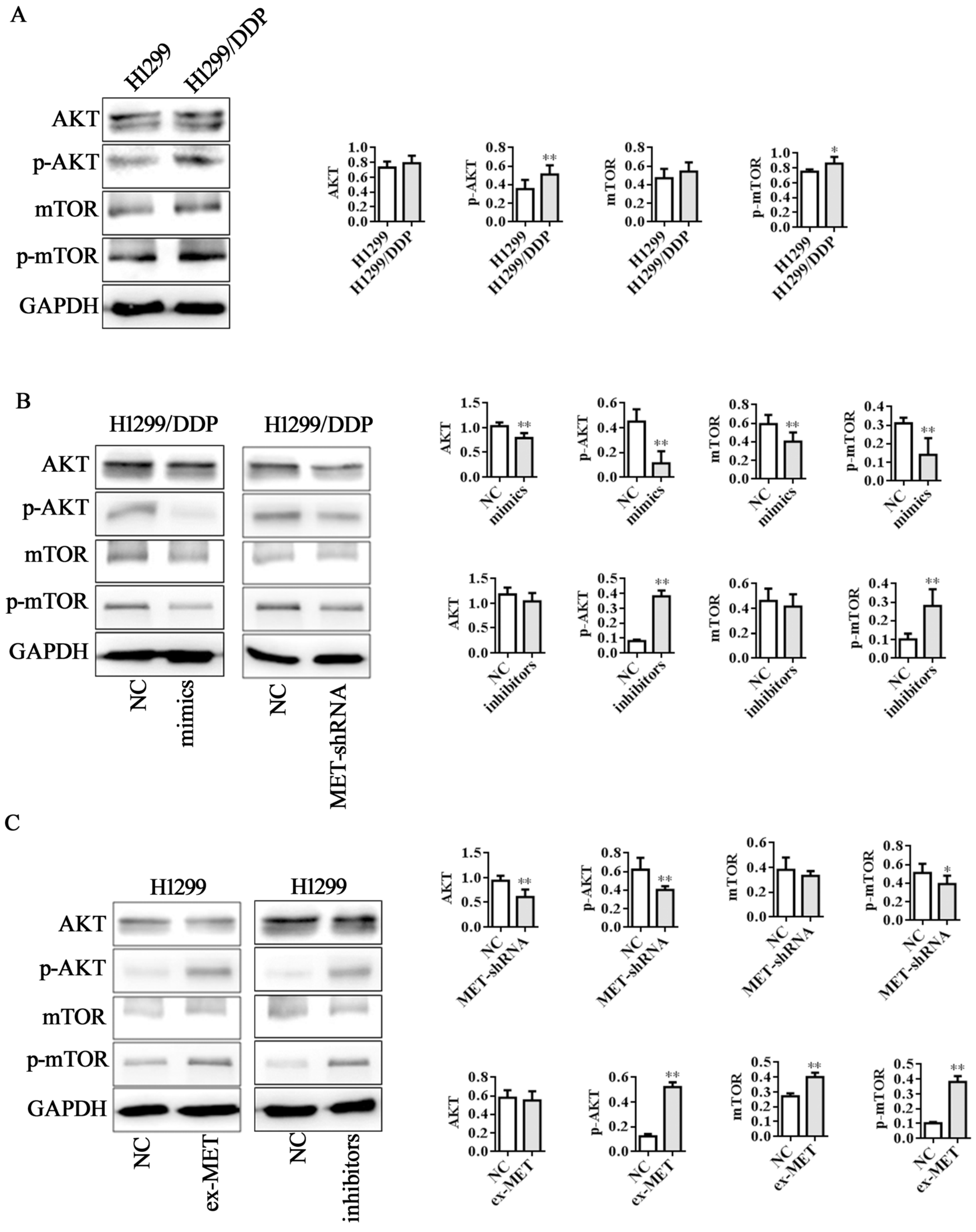

Supplementary Figure 7: miR-206/MET regulated PI3K/AKT/mTOR pathway in H1299/DDP cells. Western blotting analysis was performed to detect the protein expression of AKT, p-AKT, mTOR, p-mTOR in H1299/DDP cells and H1299 cells **A.** miR-206 mimics or MET shRNA transfected H1299/DDP cells **B.** miR-206 inhibitors or MET overexpression vectors (ex-MET) transfected H1299 cells **C.** Data are means of three separated experiments ± SD, * *P* <0.05, ** *P* <0.01 compared with their control.

Supplementary Table S1: IC 50 values for cisplatin in indicated cell lines with or without treatment

Group	Cell line	Treatment	IC 50 (μM)	*P*
1	A549	DDP	5.68±0.65	0.0003
	A549/DDP	DDP	14.78±1.18	
2	A549	DDP+inhibitor NC	6.62±0.71	0.0333
		DDP+miR-206 inhibitors	8.39±0.65	
3	A549/DDP	DDP+mimic NC	12.21±1.08	0.0007
		DDP+miR-206 mimics	5.80±0.43	
4	A549	DDP+ex-NC	5.04±0.49	0.0071
		DDP+ex-MET	6.77±0.33	
5	A549/DDP	DDP	12.82±0.55	0.0057
		DDP+ SU11274 (0.5μM)	10.58±0.46	
6	A549/DDP	DDP+NC	10.06±0.84	0.0003
		DDP+ MET shRNA	3.53±0.45	
7	A549/DDP	DDP	11.12±0.45	0.0005
		DDP+LY294002 (0.5μM)	7.78±0.31	
8	A549/DDP	DDP	11.12±0.45	0.0012
		DDP+Rapamycin (10nM)	8.31±0.39	
9	H1299	DDP	7.33±0.25	0.0000
	H1299/DDP	DDP	19.75±1.18	
10	H1299	DDP+inhibitor NC	8.93±0.77	0.0073
		DDP+miR-206 inhibitors	12.38±1.23	
11	H1299/DDP	DDP+mimic NC	18.52±1.12	0.0004
		DDP+miR-206 mimics	10.10±1.11	
12	H1299	DDP+ex-NC	8.23±0.31	0.0003
		DDP+ex-MET	13.99±0.97	
13	H1299/DDP	DDP+NC	23.07±1.44	0.0000
		DDP+ MET shRNA	9.50±1.02	

Supplementary Table S2: synthesized MET shRNA and NC oligonucleotide insert

No	Sequence
sh-MET	S: 5′- CACCGCAGTGAATTAGTTCGCTACGTTCAAGAGACGTAGCGAACTAATTCACTGCTTTTTTG-3′<br>A: 5′- GATCCAAAAAAGCAGTGAATTAGTTCGCTACGTCTCTTGAACGTAGCGAACTAATTCACTGC-3′
NC	S: 5′- CACCGTTCTCCGAACGTGTCACGTCAAGAGATTACGTGACACGTTCGGAGAATTTTTTG- 3′<br>A: 5′- GATCCAAAAAATTCTCCGAACGTGTCACGTAATCTCTTGACGTGACACGTTCGGAGAAC -3′

Supplementary Table S3: The RT and PCR primers of miR-206 used in qRT–PCR analysis

miR-206	RT primer	CTCAGCGGCTGTCGTGGACTGCGCGCTGCCGCTGAGCCACACAC
	PCR primer	F: GGCGGTGGAATGTAAGGAAG
		R: GGCTGTCGTGGACTGCG
